# Nurse-led care for the management of rheumatoid arthritis: a review of the global literature and proposed strategies for implementation in Africa and the Middle East

**DOI:** 10.1007/s00296-020-04682-6

**Published:** 2020-08-26

**Authors:** Imad Uthman, Hani Almoallim, Christopher D. Buckley, Basel Masri, Chafia Dahou-Makhloufi, Yasser El Dershaby, Nancy Sunna, Karim Raza, Kanta Kumar, Huda Abu-Saad Huijer, Nabeeha Tashkandi, Ingrid Louw, Olufemi Adelowo

**Affiliations:** 1 American University of Beirut, Beirut, Lebanon; 2 Umm Alqura University, Mecca, Saudi Arabia; 3 Institute of Inflammation and Ageing, University of Birmingham, Birmingham, UK; 4 Jordan Hospital, Amman, Jordan; 5 Bab El Oued Hospital, Algiers, Algeria; 6 Pfizer Inc, Dubai, UAE; 7 Pfizer Inc, Amman, Jordan; 8 Institute of Clinical Sciences, University of Birmingham, Birmingham, UK; 9 Hariri School of Nursing, American University of Beirut, Beirut, Lebanon; 10 King Abdulaziz Medical City for National Guard, Riyadh, Saudi Arabia; 11 Panorama Medical Centre, Cape Town, South Africa; 12 Lagos State University Teaching Hospital, Lagos, Nigeria

**Keywords:** Africa, Middle East, Nurse-led care, Rheumatoid arthritis, Rheumatology disease management, Specialist nursing

## Abstract

Globally, increasing demand for rheumatology services has led to a greater reliance on non-physician healthcare professionals (HCPs), such as rheumatology nurse specialists, to deliver care as part of a multidisciplinary team. Across Africa and the Middle East (AfME), there remains a shortage of rheumatology HCPs, including rheumatology nurses, which presents a major challenge to the delivery of rheumatology services, and subsequently the treatment and management of conditions such as rheumatoid arthritis (RA). To further explore the importance of nurse-led care (NLC) for patients with RA and create a set of proposed strategies for the implementation of NLC in the AfME region, we used a modified Delphi technique. A review of the global literature was conducted using the PubMed search engine, with the most relevant publications selected*.* The findings were summarized and presented to the author group, which was composed of representatives from different countries and HCP disciplines. The authors also drew on their knowledge of the wider literature to provide context. Overall, results suggest that NLC is associated with improved patient perceptions of RA care, and equivalent or superior clinical and cost outcomes versus physician-led care in RA disease management. Expert commentary provided by the authors gives insights into the challenges of implementing nurse-led RA care. We further report practical proposed strategies for the development and implementation of NLC for patients with RA, specifically in the AfME region. These proposed strategies aim to act as a foundation for the introduction and development of NLC programs across the AfME region.

## Introduction

The global prevalence of rheumatoid arthritis (RA) is estimated to be 0.24% and on the rise due to an aging population [1]. Although there are limited epidemiological data for Africa and the Middle East (AfME), the prevalence of RA in some AfME countries is as high as 2.54% [2–7].

A study by the American College of Rheumatology (ACR) found that the global demand for rheumatology services outstripped the supply of rheumatologists, with this imbalance projected to increase dramatically by 2030 [8]. Utilizing more non-physician service providers, such as specialist nurses, as part of a nurse-led care (NLC) model, is one suggested strategy to meet the increasing need for rheumatologists [9]. The NLC model has been defined as one in which nurses practice an extended role, assuming their own patient caseloads and providing patient services, such as treatment and monitoring, education, psychosocial support, and referral [10]. Internationally, NLC models have been used successfully in other chronic diseases, such as diabetes [11, 12], cardiovascular disease [13–15], and cancer [16]. Within the field of rheumatology, there have been specialist nurses working as part of multidisciplinary teams for over three decades in some regions [17]. Notably, in recognition of the vital role nurses can play in rheumatology care, some guidelines recommend that patients with RA have access to a nurse throughout their disease [18].

Despite global advances in rheumatology treatment, the management of RA in AfME countries remains suboptimal [19, 20], with challenges, including delayed referrals, lack of access to biologic therapies, and insufficient standardized disease assessment measures being used in clinical practice [19]. Furthermore, the burden of RA is frequently underestimated in AfME countries due to the perception that more prevalent conditions in the region (such as malnutrition, HIV, or tuberculosis) have a greater socio-economic impact, meaning that RA is not viewed as a healthcare priority [19, 21].

A lack of specialized HCPs and certified rheumatology nurse specialists also presents a major challenge to RA disease management in the AfME region, particularly in rural areas [19, 22]. Moreover, the World Health Organization (WHO) estimates that the current global shortage of nursing personnel will worsen in the AfME region by 2030 [23]. Based on the authors’ expert knowledge, the reasons for this shortage include the absence of rheumatology nurse postgraduate degrees, structured pathways for training, and career progression, including well-developed courses on advanced clinical practice, as well as a lack of understanding of training needs across the region and lack of awareness of the importance of the role of the RA nurse.

With this in mind, the objective of this literature review was to explore the importance of NLC for patients with RA in a global setting, and to combine this evidence with the authors’ expert knowledge to provide proposed strategies regarding the implementation of NLC for RA in the AfME region, specifically.

## Materials and methods

### Literature search strategy and findings

**Fig. 1:**
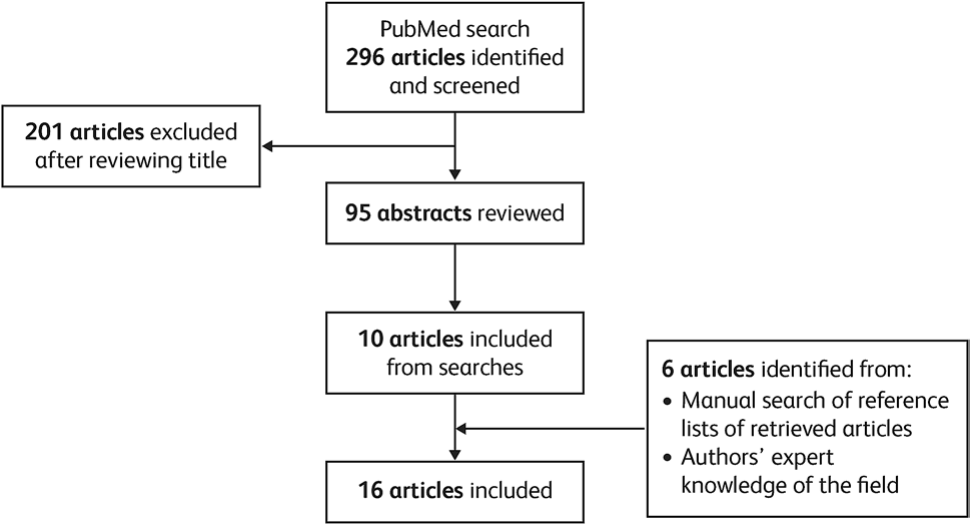
Flow chart of the narrative literature search process

We used a modified Delphi technique to draft proposed strategies, following an extensive narrative literature review. Literature searches were conducted using PubMed, incorporating studies published between January 2008 and March 2018 based on the following search terms: (“nursing” OR “nurse”) AND “care” AND (“rheumatoid arthritis” OR “rheumatic disease”). A total of 296 papers were identified, and the titles manually reviewed for relevance; the screening process is outlined in Fig. 1. Papers selected for more detailed abstract review were limited to those written in English.

A total of 16 articles were identified as being most relevant and were reviewed (Table 1) [24–39]. Relevant articles were required to include adult patients (aged ≥ 16 years) with RA. One systematic review and meta-analysis of the effectiveness of NLC versus physician-led care (PLC), representing five randomized controlled trials (RCTs) subsequently considered for inclusion, was identified. These articles were predominantly in European settings, and most were conducted in specialized rheumatology clinics. All studies (aside from the meta-analysis) were set in a single country. The role of the nurses was varied and included coordinating treatment-to-target strategies, disease activity assessment, treatment modifications, patient education, and psychosocial support (Table 1). As detailed in Table 1, four key themes (patients’ perceptions of NLC in rheumatology, clinical effectiveness of NLC in rheumatology, impact of NLC on rheumatology healthcare costs and resource use, and training and resource needs identified by rheumatology nurse specialists) were identified from the articles; these themes are explored separately in the following narrative review of the literature.

**Table 1:** Summary of the articles reviewed in this paper [24–39]

Study/article	Role of nurse	Study design	Key theme(s)	Study limitations
AMBRA				
Primdahl et al. [24]^a^	Coordination of treat-to-target strategy in diagnosed patients	24-month RCT with 287 patients with RA to evaluate patient outcomes with NLC, PLC, and shared care (no planned nurse/HCP consultations) at two rheumatology outpatient clinics in Denmark RCT primary outcome: disease activity per DAS28-CRP	Patients’ perceptions of NLC in rheumatology Clinical effectiveness of NLC in rheumatology	It was not possible to blind the treatment arm for clinicians or patients Assessment of erosion development or progression could have been more precise
Sørensen et al. [25]^a^		Cost comparison study of NLC versus PLC and shared care, based on the Primdahl et al. RCT summarized above	Impact of NLC on rheumatology healthcare costs and resource use	
Arvidsson et al. [26]	Administration of parenteral, biological, antirheumatic drugs and provision of patient education, ongoing support, and monitoring during therapy	Qualitative analysis of interviews conducted with 16 patients with RA attending a nurse-led rheumatology clinic at a hospital in Sweden	Patients’ perceptions of NLC in rheumatology	Small sample size
Bala et al. [27]	Co-ordination and assessment of treatment in diagnosed patients; patient education and support	Qualitative analysis of interviews conducted with 18 patients with RA attending one of three nurse-led rheumatology outpatient clinics in Sweden	Patients’ perceptions of NLC in rheumatology	Small sample size with male participants under-represented The first author (who conducted the interviews) was a nurse by profession which may have influenced the interview situation
de Thurah et al. [28]	N/A	Systematic review and meta-analysis of seven studies representing five RCTs that evaluated the efficacy of NLC versus PLC on disease control in 723 patients with RA^b^	Patients’ perceptions of NLC in rheumatology Clinical effectiveness of NLC in rheumatology	Interventions in the studies included in the meta-analysis were not exactly the same with respect to the frequency of follow-up and supervision provided by rheumatologists to the nurses Content in the nurse-led and physician-led follow-ups is not described in detail in the included studies and may vary based on local culture/legislation The patient populations of the studies were heterogeneous in terms of baseline disease activity and reason for dropout
Goh et al. [29]	Drug monitoring, the arrangement of basic investigations, and the education and counseling of patients were routinely performed by over 80% of respondents	Cross-sectional questionnaire study of 95 nurses employed at the time as rheumatology nurse practitioners in the UK	Training and resource needs identified by rheumatology nurse specialists	Potential for selection bias in the sampling technique as the target population was identified using the British Health Professions in Rheumatology handbook
Koksvik et al. [30]^a^	Disease assessment, patient education, and counseling	21-month RCT with 68 patients with IA (RA, AS, PsA, JIA, or undifferentiated polyarthritis) to evaluate the effect of NLC versus PLC in a rheumatology outpatient setting at a hospital in Norway RCT primary outcome: patient satisfactory per LSQ	Patients’ perceptions of NLC in rheumatology Clinical effectiveness of NLC in rheumatology	Patients had a variety of diagnoses and varying levels of disease activity, and the study was not powered to investigate subgroup differences. Findings are only applicable to patients with low disease activity Despite being blinded to randomization, patients assigned to NLC may have known their nurse from prior visits
Larsson et al.				
Larsson et al. [31]^a^	Monitoring patients receiving biologic therapy	12-month RCT with 107 patients with CIA (RA, PsA, undifferentiated arthritis, or undifferentiated SpA) receiving biological therapy to evaluate clinical outcomes with NLC versus PLC at a rheumatology clinic in Sweden RCT primary outcome: disease activity per DAS28	Clinical effectiveness of NLC in rheumatology	Short time-perspective
Larsson et al. [32]		Cost comparison study of NLC versus PLC, based on the Larsson et al. [31] RCT summarized above	Impact of NLC on rheumatology healthcare costs and resource use	
Muñoz-Fernández et al. [33]	Not specified	12-month observational study and economic evaluation of NLC versus PLC with patients with RA (*n* = 302) and AS (*n* = 91) attending rheumatology clinics in Spain	Clinical effectiveness of NLC in rheumatology Impact of NLC on rheumatology healthcare costs and resource use	Short time-perspective A higher proportion of patients receiving NLC received bDMARD treatment versus those receiving PLC, which may have influenced clinical outcomes
Ndosi et al. [34]^a^	Administration, monitoring, and coordination of treatment; patient education and support	12-month, multicenter RCT with 181 patients with RA attending one of ten rheumatology outpatient clinics to evaluate clinical- and cost-effectiveness of NLC versus PLC in the UK RCT primary outcome: disease activity per DAS28	Patients’ perceptions of NLC in rheumatology Clinical effectiveness of NLC in rheumatology Impact of NLC on rheumatology healthcare costs and resource use	Short time-perspective Only 73.5% of randomly assigned patients had complete data for the primary endpoint at all follow-up visits Information on interventions was quantitative rather than qualitative detail of what was involved Generalizability of these findings is limited; NLC effectiveness is likely to be related to how well the model is developed in individual settings
Riley et al. [35]	Not specified	Quantitative analysis of surveys conducted with 2338 nurse practitioners working with rheumatology patients in a primary care setting in the US	Training and resource needs identified by rheumatology nurse specialists	As respondents were self-selected, they may reflect an atypical sample with a greater desire and need for education/professional support compared with the overall nurse practitioner population Respondents’ subjectivity may have led to a response bias Nurse practitioners worked in a primary care setting (rather than a rheumatology-specific setting)
Sjö et al. [36]	Disease activity assessment, treatment monitoring, and adjustment	Qualitative analysis of surveys conducted with 15 patients with RA attending a nurse-led rheumatology clinic at a hospital in Sweden over 12 months	Patients’ perceptions of NLC in rheumatology	The interviewer was a nurse by profession which may have influenced the interview situation Small sample size
Solomon et al. [37]	Disease activity assessment; treatment prescription and monitoring; arranging laboratory/radiology tests	An observational cohort study of 301 patients with RA attending seven rheumatology practices in the US to compare care provided in practices with nurse practitioners or physician assistants with rheumatologists versus rheumatologists only over a 24-month follow-up period	Clinical effectiveness of NLC in rheumatology	Small sample size Limited data collection Disease activity measures were missing for some patient visits Disease activity measurements were conducted by various providers across numerous practices without standardized teaching, which may have introduced misclassification
Vinall-Collier et al. [38]	Not specified	Multicenter mixed methods study of patient/HCP interactional style in nine nurse-led and physician-led rheumatology clinics in the UK	Patients’ perceptions of NLC in rheumatology	Small sample size All HCPs and patients were self-selecting Some interviews were short (median = 15 min)
Wang et al. [39]	Disease activity assessment; treatment monitoring and prescription; patient education and support; ordering x-rays or blood tests; managing admissions or referrals to a rheumatologist or other HCP	12-month RCT with 214 patients with RA to evaluate the clinical- and cost-effectiveness of NLC versus PLC at a hospital in China RCT primary outcome: disease activity per DAS28	Clinical effectiveness of NLC in rheumatology Impact of NLC on rheumatology healthcare costs and resource use	Short time-perspective As this was designed as a preliminary study, findings cannot be generalized to the overall Chinese population

*AS* ankylosing spondylitis, *CIA* chronic inflammatory arthritis, *CRP* C-reactive protein, *DAS28* Disease Activity Score in 28 joints, *HCP* healthcare professional, *IA* inflammatory arthritis, *JIA* juvenile idiopathic arthritis, *LSQ* Leeds Satisfaction Questionnaire, *N/A* not applicable, *NLC* nurse-led care, *PLC* physician-led care, *PsA* psoriatic arthritis, *RA* rheumatoid arthritis, *RCT* randomized controlled trial, *SpA* spondyloarthritis, *UK* United Kingdom, *US* United States

^a^RCTs included in the de Thurah et al. [28] meta-analysis, reviewed in this paper

^b^Two RCTs included in the meta-analysis are not evaluated individually in this paper as they were superseded by later studies with similar endpoints: Hill et al. [40] and Primdahl et al. [41] (see Primdahl et al. [24] for the latter RCT)

The findings of the narrative review were summarized and presented to all authors. The author group comprised experts in rheumatology and nursing from different countries in the AfME region (Algeria, Jordan, Lebanon, Nigeria, Saudi Arabia, South Africa, and UAE) and from the UK. Virtual open discussions were held between authors. All authors’ viewpoints and remarks were collected, summarized, and presented for further verification and adjustment. A consensus was developed by each author reviewing, adjusting, adding, deleting, combining, reforming, and approving proposed strategies. Each author participated in wording each proposed strategy.

## Results

### Patients’ perceptions of NLC in rheumatology

The literature search identified eight articles that explored the perceptions of NLC by patients with RA (Table 1).

As reported in interviews/surveys conducted with patients with RA in a Swedish rheumatology clinic, nurse-led management, information, and support was shown to be important in increasing patients’ sense of empowerment [26]. Nurses instilled feelings of security, trust, hope, and confidence [27, 36], and patients reported that they found it easier to discuss queries with nurses than with physicians [26]. Interestingly, in a 2016 study of the interactional style between patients and HCPs, patients with RA were seen to initiate more ‘personal talk’ and provide more unprompted information relevant to their care in nurse-led consultations, compared with physician-led consultations [38]. Therefore, it is unsurprising that the same study found nurses to engage in significantly more relationship building with their patients, compared with physicians [38]. In terms of the qualities that patients valued in their nurses, regular accessibility [26, 27, 36], competency in their disease and treatment knowledge [36], giving clear and meaningful explanations [27], and the provision of a patient-centered/holistic approach [26, 36] were mentioned consistently.

A 2017 systematic review and meta-analysis of RCTs concerning the efficacy of, and patient satisfaction with, NLC had mixed results with respect to the latter: no significant differences in patient satisfaction with NLC versus PLC after a 1-year follow-up were reported (data from four RCTs) [28]. However, after a 2-year follow-up, significantly greater patient satisfaction was observed with NLC versus PLC (*P* < 0.05; data from two RCTs up to this time point) [28]. Specifically, one of these RCTs reported that patients with RA receiving NLC had significantly increased self-efficacy (defined as patients’ belief in their ability to perform specific tasks or behaviors to cope with their RA), confidence, and satisfaction at the 2-year follow-up versus patients receiving PLC [24]. Two studies in the meta-analysis used the Leeds Satisfaction Questionnaire (LSQ), a validated, self-administered questionnaire in which patients respond to a series of statements using a five-point Likert scale to assess their satisfaction levels across six subscales: general, information, empathy, technical, attitude, access [30, 34]. Results using this tool were mixed. Koksvik et al. reported significantly greater patient satisfaction across all subscales of the LSQ for patients with inflammatory arthritis (IA; RA, ankylosing spondylitis [AS], psoriatic arthritis [PsA], juvenile idiopathic arthritis [JIA], or undifferentiated polyarthritis) receiving NLC versus PLC at 9- and 21-month follow-up (primary endpoint; all *P* < 0.001, excepting general satisfaction at 9-month follow-up for which *P* < 0.05) [30]. However, in Ndosi et al., while general satisfaction scores were significantly higher with NLC versus PLC at 26-week follow-up (*P* < 0.05), no numerical differences in scores between patient groups were reported across the remaining LSQ subscales [34]. At 52-week follow-up, general satisfaction scores were similar to NLC versus PLC; as at the earlier time point, there were no numerical differences across the remaining LSQ subscales between patient groups [34].

### Clinical effectiveness of NLC in rheumatology

The clinical effectiveness of NLC for patients in rheumatology care was evaluated in eight articles identified in the literature search (Table 1). A 2017 systematic review and meta-analysis of RCTs by de Thurah et al. reported no differences in disease activity data (per Disease Activity Score in 28 joints, erythrocyte sedimentation rate/C-reactive protein [DAS28-4 [ESR/CRP]]) between NLC versus PLC at 1-year follow-up (data from four RCTs), and a statistically significant (*P* < 0.05), but not clinically relevant, the difference in disease activity favoring NLC at 2-year follow-up (data from two RCTs up to this time point) [28]. Of the two RCTs in the de Thurah et al. paper that reported disease activity at 1-year follow-up only, Ndosi et al. found that improvements in RA disease activity (primary endpoint; per DAS28) over 52 weeks for British patients receiving NLC were non-inferior to improvements for patients receiving PLC [34]. Similarly, a 12-month RCT in Sweden, as reported by Larsson et al. found that improvements in disease activity (primary endpoint; per DAS28-4 [ESR] and DAS28-4 [CRP]) were non-inferior in patients with stable chronic IA (CIA; RA, PsA, undifferentiated arthritis, or undifferentiated spondyloarthritis [SpA]) undergoing biologic therapy who had one of two annual rheumatologist monitoring visits replaced by a nurse-led monitoring visit, versus patients who saw a rheumatologist at both visits [31]. Of the two RCTs evaluated by de Thurah et al. that reported both 1- and 2-year disease activity data, Primdahl et al. reported no significant difference in disease activity (primary endpoint; per DAS28-4 [CRP]) in patients with RA receiving NLC versus PLC at 12 months, but found NLC to be significantly superior at 24 months (*P* = 0.049) [24]. Koksvik et al. found that improvements in disease activity (per DAS28-4 [ESR]) were significantly greater in patients with IA (RA, AS, PsA, JIA, or undifferentiated polyarthritis) receiving NLC versus PLC at 9-month follow-up (between-group difference in DAS28 [ESR] 0.45; *P* = 0.03), but no significant difference was seen at 21-month follow-up (between-group difference in DAS28 [ESR] 0.31; *P* = 0.15) [30].

Of the three further articles that explored the impact of NLC on disease activity (per DAS28), Muñoz-Fernández et al. found that there were no significant differences in RA disease activity with NLC versus PLC at 12-month follow-up (mean DAS28 of 2.7 vs. 2.8, respectively; *P* = 0.274) [33]. Similarly, across seven rheumatology practices in the US, no differences in changes in disease activity (per DAS28, Clinical Disease Activity Index [CDAI], and Routine Assessment of Patient Index Data 3 score [RAPID3]) were observed between patients with RA treated by nurse practitioners or physician assistants versus those treated by rheumatologists only over 2 years [37]. However, a 12-month RCT conducted in China found that improvements in RA disease activity (per DAS28) were significantly greater in patients receiving NLC versus PLC throughout the study period (primary endpoint; *P* < 0.001) [39].

The impact of NLC on a range of patient-reported outcomes (PROs) in patients with RA was explored in six studies, with mixed results [24, 30, 31, 33, 34, 39]. Wang et al. found that improvements in pain, fatigue, and stiffness were significantly greater with NLC versus PLC [39], while Muñoz-Fernández et al. reported significantly greater improvements in physical function and health-related quality of life (HRQoL) with NLC versus PLC [33]. In contrast, several studies reported no significant differences in improvements across PROs, including pain [24, 30, 31], physical function [24, 31], HRQoL [30], and fatigue [24, 30] in patients receiving NLC versus PLC. Finally, Ndosi et al. found that improvements in pain, physical function, fatigue, and stiffness in patients receiving NLC were non-inferior to improvements in patients receiving PLC [34].

### Impact of NLC on rheumatology healthcare costs and resource use

The impact of NLC on rheumatology healthcare costs and resource use was assessed in five studies (Table 1). A 12-month RCT in Sweden of patients with stable CIA (RA, PsA, undifferentiated arthritis, or undifferentiated SpA) undergoing biologic therapy found that total annual rheumatology care costs per patient (including fixed monitoring, variable monitoring, rehabilitation, specialist consultations, radiography, and pharmacological therapy) were significantly lower for patients who had one of two annual rheumatologist monitoring visits replaced by a nurse-led monitoring visit versus patients who saw a rheumatologist at both visits (13% reduction in costs; *P* < 0.01) [32]. When annual resource use was investigated further, there were no significant differences between NLC and PLC for costs related to additional phone calls or visits to nurses/rheumatologists, rehabilitation, physiotherapy, occupational therapy, psychosocial treatment, specialist consulting, or radiography identified; while costs related to pharmacological therapy and additional blood tests were significantly higher with PLC versus NLC (*P* < 0.05) [32]. A 12-month RCT found that unplanned hospitalizations or additional clinic visits were less common in Chinese patients with stable RA receiving NLC versus PLC, with overall costs related to RA treatment, laboratory tests, radiography, steroid injections, and daycare admissions significantly lower with NLC versus PLC [39]. In addition to the reduced hospital visits observed with NLC, authors hypothesized that cost differences might also be due to the lower number of prescriptions, laboratory tests, and radiological investigations ordered for the NLC- versus PLC-recipient groups [39]. A 12-month RCT conducted in the UK also reported numerically lower rates of unplanned hospital admissions or visits to the accident and emergency department or general practitioner surgery with NLC versus PLC. However, differences in mean overall costs for RA care (including costs of clinic and specialist visits, community care, investigations, hospitalization, and medications) were not statistically significant between NLC and PLC groups, despite consultation costs for NLC being significantly lower versus PLC (*P* < 0.001) [34].

Similarly, a 2016 observational study conducted in Spain that evaluated the economic impact of NLC versus PLC in the treatment of patients with RA and AS reported that, while the costs of consultations at healthcare clinics were significantly lower with NLC versus PLC (*P* = 0.001), overall, there were no significant differences in total annual healthcare costs or indirect costs (work, travel, or carer-related) between the two systems [33]. Of the studies that conducted cost-effective analyses, Ndosi et al. found NLC to be cost-effective in comparison with PLC when health benefit was measured as a change from baseline in disease activity (primary endpoint; per DAS28), but not when health benefit was measured as quality-adjusted life years (QALY) [34]. However, a 24-month RCT in Denmark, in which health benefit was measured as QALY, found NLC to be cost-effective in the management of RA in comparison with both PLC and shared care (no planned nurse or rheumatologist consultations) [25].

### Training and resource needs to be identified by rheumatology nurse specialists

Two articles provided considerations for training and resource needs identified by rheumatology nurse specialists (Table 1). While evidence suggests that NLC is generally associated with improved clinical and cost outcomes in the management of RA, there could be the potential to further optimize NLC by meeting additional training and resource needs identified by nurses working in rheumatology. A 2006 questionnaire administered to 95 nurse practitioners working in rheumatology departments in the UK identified the following factors that could enhance their role [29]: attendance at postgraduate courses and obtaining further qualifications; active participation in the delivery of medical education; training in practical procedures such as intra-articular injections; protected time and resources for audit and research; formal training in counseling; and implementation of nurse prescribing. Many of these factors were linked with attributes that nurses identified as being necessary for their competency (such as knowledge and understanding of rheumatic diseases and drug therapy). More recently, an extensive 2017 survey of 2338 nurse practitioners working with rheumatology patients in a primary care setting in the US identified the following resource and training needs to optimize NLC [35]: provision of an RA medication chart with indications/contraindications, adverse events, and monitoring advice to help determine the best course of treatment; an RA assessment tool for better management of patients; further education on the long-term efficacy and safety of RA medications; and access to academic conferences, events, peer-reviewed journals, and online forums or educational tools to facilitate the exchange of educational information with other HCPs.

### Expert commentary: challenges around the implementation of NLC in the AfME region

An insight into the challenges facing the implementation of NLC in the AfME region was provided by the authors.

The wealth disparity across the AfME region impacts significantly on available healthcare resources, and limits opportunities for implementing and expanding multidisciplinary teams. The most recent data from the World Bank reports that health expenditure per capita, per year, ranges from US dollars (USD) 198.00 in sub-Saharan Africa to USD 1287.69 in the Middle East and North Africa [42]. With the health system chronically underfunded in numerous countries across the AfME region, there are shortages of nurses, with an unbalanced distribution between urban and rural areas. Also, negative cultural perceptions of the nursing profession, long working hours, and relatively low pay have further contributed to a nursing shortfall. As such, in areas with a limited nursing workforce, it may not be possible for nurses to focus on a single specialty. Furthermore, although recognition of nurse specialization is increasing, there remains the challenge of nurses emigrating from sub-Saharan African countries to areas where they receive greater appreciation and remuneration (author opinion).

In the opinion of the authors, NLC may be able to help overcome some of the particular challenges facing rheumatology care in the AfME region. For example, studies have shown that in Saudi Arabia and some African countries, the diagnostic delay of RA is long, between 2.5 and > 4 years [43–45], thereby preventing early access to treatment before irreversible joint damage. As a result, when patients are eventually diagnosed with RA, they often already have erosive disease or high disease activity. Access to NLC may relieve the burden on rheumatologists and allow quicker access to a specialist HCP. However, even after diagnosis, patients with RA are often undertreated in the region, with access to therapies dependent on the nation’s healthcare system, drug availability, and economic status [46]. Lack of access to biologic therapies has been highlighted as a particular challenge to the implementation of standardized European League Against Rheumatism (EULAR) treatment guidelines in Africa [19]. At the same time, a recent study conducted in five Arab countries (Jordan, Lebanon, Qatar, Saudi Arabia, and UAE) found significant differences in the use of biologic therapies between countries [47]. Patient educational deficits around the efficacy of treatments and the perception of RA as an incurable disease may also pose additional barriers to treatment acceptance. Specialized nurses with an in-depth knowledge of the available treatment pathways could enhance patient education. Furthermore, they could act as advocates for improved patient treatment and promote the wider use of more advanced therapies across the field of rheumatology (author opinion).

### Expert commentary: proposed strategies for the implementation of NLC in the AfME region

The evidence discussed across each of the four key themes identified above was considered by the authors, and based on the expert knowledge of the authors, we propose a series of strategies, shown in Table 2 [48–53], for the implementation of NLC in the AfME region.

**Table 2:** Proposed strategies for the implementation of NLC in the AfME region [48–53]

	1. Define what NLC models would encompass across the AfME region	
a. Tailored models should be developed at a national level, adapted to meet the needs of individual countries, with consideration of the burden of disease and health resources in each country		
b. Consider how NLC could complement, and be integrated within, current pathways of care. A model of clinical nurse specialist and physician collaboration in the care of colorectal and stoma patient populations in Saudi Arabia, that considers patient population needs and local healthcare culture [48], could be used as a template		
c. Seek endorsement of NLC models by regional and country-specific health regulators, guideline committees, and policymakers		

	2. Promote rheumatology as a specialism and develop formal pathways for rheumatology nurse certification	
a. Advocate and promote the value of the rheumatology specialism through the undergraduate nursing curriculum, development of which could be led by relevant universities. MSK modules should be incorporated to equip non-specialist nurses better to play an active role in RA care		
b. Advocate for the launch of Clinical Nurse Specialists in Rheumatology postgraduate training pathways, up to Masters level, in AfME nursing schools. These programs would have selection criteria, including a minimum of 4 years’ experience. Nurses would be trained in the diagnosis and assessment of RA through mentoring/shadowing schemes with rheumatologists in the clinic. State board examinations would be performed to certify advanced nurse specialists in rheumatology. By credentialing nurses, this would ultimately empower them to have the autonomy and accountability to lead RA care		
c. National or regional nursing councils/commissions, developed in collaboration with specialized physicians and nursing leaders, could be equipped to certify rheumatology nurses. Notably, the Jordanian Nursing Council has developed a Directorate of Specialization, which has determined a framework for nursing specialization. Furthermore, the Saudi Arabia Commission for Health Specialties has approved postgraduate nursing diplomas for a range of specialties. The authors recommend that these existing programs should be further expanded to offer a diploma for chronic diseases that would encompass diabetes, rheumatology, and renal diseases. These programs could act as templates for similar models to be developed		
d. Provide scholarship opportunities and stipends for those undertaking further study in rheumatology programs, to increase uptake		

	3. Provide accessible resources and training programs for nurses to specialize in rheumatology	
a. Develop educational materials/handbooks outlining standardized guidelines for nurses managing patients with RA, and taking into account the health resources and socioeconomic situations of each country		
b. Promote and increase awareness of national rheumatology societies as a platform for sharing educational resources and initiatives. These societies could deliver rheumatology training workshops to nurses and could help develop accessible resources such as e-learning courses to address challenges with respect to access to face-to-face training. Frameworks for these programs could be based on successful, online rheumatology programs currently offered and/or endorsed by the ACR’s Association of Rheumatology Health Professionals, the Rheumatology Nurses Society, and the Pan-American League of Associations for Rheumatology		
c. Develop fellowship schemes to enable HCPs to visit key centers where advanced rheumatology nurses practice, in order for HCPs to observe and learn from NLC in practice		

	4. Improve RA disease management by expanding the role of nurses	
a. Advocate for the establishment of nurse-led clinics to lead the day-to-day management (encompassing patient education, referrals, treatment, and monitoring) of RA. Crucially, these clinics should be part of the public healthcare system. A mentorship program could be led and supervised by physicians and nursing leaders to ensure that the care provided is within nurses’ scope of practice. In addition, mandatory patient follow-ups with the consultant physician should be conducted after a certain number of visits to ensure sufficient care is being delivered		
b. Implement nurse-led triage systems in the clinic, with adequate, credentialed training, through mentoring/shadowing schemes with rheumatologists, to improve diagnosis and referral times		
c. Establish nurse-led patient support programs to improve patients’ knowledge and confidence around their RA treatment. Support should be secured from local healthcare authorities by highlighting the advantages for the healthcare economy, as well as improved care for patients. Notably, in Lebanon, patient support groups are relatively common and readily accepted in several fields, such as cancer. In RA, a national patient support program exists in Saudi Arabia, which provides patient counseling regarding the use of biological agents [53]; this could be used as a template for similar nurse-led programs		
d. Establish nurse-led telephone services and/or digital communication platforms to enhance continuity of care and provide ongoing support to patients with RA. Patients could be provided with contact details so they can reach nurses for assistance. Such services would be particularly valuable in remote areas of the region, where lack of local services, long travel times, and lack of infrastructure can limit patients’ contact with their RA HCPs		

		5. Educate patients on the role of nurses in RA disease management
a. A formal study should be conducted to assess variations in patients’ perceptions of nurses. Patient education strategies should then be shaped in line with these regional perceptions to ensure patients understand the value of, and accept, rheumatology nurse specialists as a key part of their care		
b. Advocate for the delivery of government-sponsored public health outreach programs to promote the benefits of NLC. Activities could encompass social and print media campaigns for a lay audience		
c. The concept of NLC should first be introduced to patients by rheumatologists in the presence of rheumatology nurse specialists to ensure patients feel confident that this is a legitimate and respected pathway of care		

		6. Conduct formal studies to evaluate the impact of NLC across the AfME region
a. Conduct research into the outcomes and experiences with NLC in the management of other diseases in the AfME region to inform NLC for RA		
b. Following the implementation of NLC in rheumatology services, conduct local studies (encompassing RCTs or qualitative research studies) to evaluate the effectiveness of NLC versus previously available treatment options. These findings could then be used to shape the future development of NLC in the region and promote the benefits of NLC to both local patients and healthcare authorities		

Proposed strategies are based on the authors’ knowledge of the AfME region in general. They may not be specific to individual countries

*ACR* American College of Rheumatology, *AfME* Africa and the Middle East, *HCP* healthcare professional, *IA* inflammatory arthritis, *MSK* musculoskeletal, *NLC* nurse-led care, *RA* rheumatoid arthritis, *RCT* randomized controlled trial

## Discussion

In summary, the articles identified in this literature search provide compelling support for NLC in RA disease management. The roles of nurses in the NLC examples reviewed were varied. In addition to monitoring disease activity and treatment, NLC facilitates patient education and support, and nurses are able to coordinate multidisciplinary care. Patient perceptions of NLC are generally positive, and reported as better than or comparable with PLC, with no real patient-reported barriers to care identified.

Furthermore, there is evidence that NLC enhances clinical outcomes in patients, with most evidence focusing on disease activity (per DAS28). At the same time, from a health economics perspective, NLC is associated with better or comparable cost-effectiveness versus PLC. Finally, rheumatology nurses have important suggestions regarding the enhancement of NLC. Limitations acknowledged by the authors of the articles include the need for more data in this area from a greater number of patients, within which subgroups (for example, by disease type and activity) can be assessed over prolonged periods. Increased use of NLC could provide such data.

Overall, given the clear benefits associated with NLC, it is justified to propose strategies for the implementation of this practice in the AfME region. The evidence discussed across each of the four key themes identified above was considered by the authors and allowed the proposal of strategies for NLC programs across the AfME region.

There was no scoring system given for each author to determine the weight of each proposed strategy. Rather, the proposed strategies were collectively agreed upon using a modified Delphi technique. Overall, the proposals represent important strategies to be considered across the AfME region for the development of NLC programs. However, the value and importance of some strategies may vary by country, based on differences in their economic and educational systems. As such, it was not appropriate to assign a weight to each proposed strategy.

The findings of this narrative literature review are limited by the inclusion of articles from a small geographic area, which may limit the generalizability of the findings. In addition, the application of these strategies may be limited by the legal requirements, differences in clinical practice, cost, cultural differences, and patient perception in each country. Nevertheless, these proposed strategies aim to act as a foundation for the introduction and development of NLC programs across the AfME region.

## Conclusions

NLC in RA disease management has been shown to have positive impacts on patients’ perceptions of their treatment, clinical outcomes, and healthcare costs. Despite these benefits, there is a lack of rheumatology nurse specialists across AfME, a region where there is low public disease awareness and inadequate treatment for RA. The proposed strategies presented in this paper aim to act as a foundation for the development of NLC programs across the AfME region. Looking to the future, specialist nursing could move away from focusing on a specific disease, such as RA, and instead move towards working across disease taxonomies, such as immune-mediated rheumatic disorders [54]. It is the expert opinion of the authors that such a move could further enhance cost-effectiveness in training and establishing nurses to lead care in a range of clinical settings across the AfME region.

## Abbreviations

ACR: American College of Rheumatology
AfME: Africa and the Middle East
AS: Ankylosing spondylitis
CI: Confidence interval
CRP: C-reactive protein
DAS28: Disease Activity Score in 28 joints
ESR: Erythrocyte sedimentation rate
EULAR: European League Against Rheumatism
HCP: Healthcare professional
HRQoL: Health-related quality of life
IA: Inflammatory arthritis
JIA: Juvenile idiopathic arthritis
LSQ: Leeds Satisfaction Questionnaire
MSK: Musculoskeletal
NLC: Nurse-led care
PLC: Physician-led care
PRO: Patient-reported outcome
PsA: Psoriatic arthritis
QALY: Quality-adjusted life years
RA: Rheumatoid arthritis
RCT: Randomized controlled trial
SpA: Spondyloarthritis
UAE: United Arab Emirates
UK: United Kingdom
US: United States
USD: United States dollars
WHO: World Health Organization
